# Immune Responses after Heavy Alcohol Consumption: Cytokine Concentrations in Hangover-Sensitive and Hangover-Resistant Drinkers

**DOI:** 10.3390/healthcare9040395

**Published:** 2021-04-01

**Authors:** Aurora JAE van de Loo, S. Jorinde Raasveld, Anna Hogewoning, Raymond de Zeeuw, Else R Bosma, Noor H Bouwmeester, Melanie Lukkes, Karen Knipping, Marlou Mackus, Aletta D Kraneveld, Karel A Brookhuis, Johan Garssen, Andrew Scholey, Joris C Verster

**Affiliations:** 1 Division of Pharmacology, Utrecht Institute for Pharmaceutical Sciences, Utrecht University, 3584CG Utrecht, The Netherlands; a.j.a.e.vandeloo@uu.nl (A.J.v.d.L.); jorinde.raasveld@gmail.com (S.J.R.); a.hogewoning@students.uu.nl (A.H.); raymond_sb3@live.nl (R.d.Z.); bosmaer@gmail.com (E.R.B.); nh.bouwmeester@gmail.com (N.H.B.); melanielukkes@hotmail.com (M.L.); m.mackus@uu.nl (M.M.); a.d.kraneveld@uu.nl (A.D.K.); j.garssen@uu.nl (J.G.); 2 Global Centre of Excellence Immunology, Nutricia Danone Research, 3584CT Utrecht, The Netherlands; karen.knipping@danone.com; 3 Faculty of Behavioral and Social Sciences, Groningen University, 9700AB Groningen, The Netherlands; k.a.brookhuis@rug.nl; 4 Centre for Human Psychopharmacology, Swinburne University, Melbourne, VIC 3122, Australia; andrew@scholeylab.com

**Keywords:** alcohol, hangover, immune system, cytokine, hangover severity

## Abstract

This study investigated immunological changes during an alcohol hangover, and the possible difference between hangover-resistant and hangover-sensitive drinkers in terms of immune reactivity. Using a semi-naturalistic design, N = 36 healthy social drinkers (18 to 30 years old) provided saliva samples on a control day (after drinking no alcohol) and on a post-alcohol day. Hangover severity was rated directly after saliva collection. Cytokine concentrations, interleukin (IL)-1β, IL-6, IL-8, IL-10 and tumor necrosis factor (TNF)-α, and hangover severity were compared between both test days and between hangover-sensitive and -resistant drinkers. Data from N = 35 drinkers (17 hangover-sensitive and 18 hangover-resistant) were included in the statistical analyses. Relative to the control day, there were significant increases in saliva IL-6 and IL-10 concentrations on the post-alcohol day. No significant differences in cytokine concentrations were found between hangover-sensitive and hangover-resistant drinkers, nor did any change in cytokine concentration correlate significantly with hangover severity. In line with previous controlled studies assessing cytokines in blood, the current naturalistic study using saliva samples also demonstrated that the immune system responds to high-level alcohol intake. However, further research is warranted, as, in contrast to previous findings in blood samples, changes in saliva cytokine concentrations did not differ significantly between hangover-sensitive and hangover-resistant drinkers, nor did they correlate significantly with hangover severity.

## 1. Introduction

Alcohol hangover refers to the combination of negative mental and physical symptoms which can be experienced after a single episode of alcohol consumption, starting when blood alcohol concentration (BAC) approaches zero [1,2]. A hangover is characterized by a variety of symptoms [3,4] that may impair daily activities such as job performance or driving a car [5,6]. Being hungover also negatively influences mood [7,8].

Despite these functional consequences, the pathology of the alcohol hangover has received relatively little research attention. The immune system has been proposed to play a role in the pathology of the alcohol hangover [9,10]. It is hypothesized that cytokines are released as a response to alcohol intake and subsequently interact with the central nervous system (CNS), contributing to the various next-day symptoms collectively called the hangover state. The immune system communicates with the CNS [11,12]. Peripherally released cytokines influence CNS functioning, either through the endocrine pathway or via the vagus nerve, resulting in upregulation of central cytokine production [13,14,15]. In healthy volunteers, acute blood cytokine concentration changes have been demonstrated, starting 20-min post-alcohol-consumption [16,17]. It is hypothesized that these changes in immune response may influence the presence and/or severity of the next-day alcohol hangover. 

To date, few studies have investigated immune function changes during alcohol hangover. Kim et al. [18] collected blood samples from 20 male subjects (all with a history of hangovers after drinking) on an alcohol-free day and the following day after an evening of consuming soju, an alcoholic beverage made from rice, barley, or wheat. Blood samples were collected prior to drinking and then the morning following alcohol administration. The blood samples were stimulated by adding phytohemagglutinin (PHA) before cytokine concentration assessments. Of the seven cytokines measured, there were significant elevations in interleukin (IL)-10, IL-12 and interferon-gamma (IFN-γ), but not in IL-1β, IL-4, IL-6, or tumor necrosis factor-alpha (TNF-α). Kim et al. found significant correlations between blood ΔIL-12 and ΔIFN-γ concentrations and changes in overall hangover severity. Significant correlations with subjective hangover scale scores were found for ΔIFN-γ, and significant correlations with somatic hangover scale scores were found for ΔTNF-α and ΔIL-12, suggesting a meaningful contribution to hangover symptomatology [18]. 

Kim et al. [19] examined the effects of Hovenia dulcis Thunb fruit extract on alcohol hangover. In a double-blind, placebo-controlled study, blood samples were taken at baseline and 1, 4, and 12 h after alcohol consumption (Korean soju, containing 50 g alcohol). TNF-α, IFN-γ, IL-6, IL-10, and IL-12 concentrations were determined. Kim et al. [19] found significant correlations between blood IL-6 and IL-10 concentrations and hangover severity. 

Mammen et al. [20] examined the effects of clove bud polyphenols (Clovinol, 250 mg) on hangover severity. In a double-blind, placebo-controlled study, alcohol (1 g/kg) was administered to 16 male social drinkers and blood samples were taken at 0, 0.5, 2, 4, and 12 h after treatment. Alcohol significantly increased blood concentrations of C-reactive protein and IL-6. Treatment with Clovinol significantly reduced blood concentrations of C-reactive protein and IL-6 and significantly reduced overall hangover severity. No correlations between hangover severity and cytokine concentrations were presented, but the authors reported that the observed elevation of IL-6 was highest among subjects who reported severe hangover symptoms. 

Whereas most drinkers experience hangovers after heavy drinking, approximately 10–25% of social drinkers claim to be hangover-resistant, despite consuming large quantities of alcohol [21,22,23,24]. The symptoms reported by these hangover-resistant drinkers, if any, tend to be limited to mild drowsiness-related descriptors [25]. Comparing hangover-sensitive and hangover-resistant drinkers may increase our knowledge of the underlying pathology of the alcohol hangover. For example, immune reactivity may differ between the two groups, which could explain the absence of hangover symptoms in the resistant group. To date, no direct comparisons between hangover-sensitive and hangover-resistant individuals comparing biomarkers of immune functioning have been published. 

The current data on immune activation after heavy alcohol consumption come from controlled studies. However, in real life, often, higher amounts of alcohol are consumed than those administered in controlled experiments [25]. To enhance ecological validity, the current study had a semi-naturalistic design, in which the investigators did not interfere with the drinking session. The primary aim of the study was to investigate saliva cytokine concentrations, during a post-alcohol day and a control day. A secondary aim was to directly compare hangover-sensitive drinkers with hangover-resistant drinkers. It was hypothesized that, compared with hangover-sensitive drinkers, hangover-resistant drinkers would display a reduced immune response. It was further hypothesized that hangover severity correlates significantly with the magnitude of the changes in cytokine concentrations.

## 2. Materials and Methods

This was a semi-naturalistic study, consisting of a training day and two test days, with approximately one week scheduled between the test days (for a full description, see [25]). Participants consumed alcohol at a venue of choice, with corresponding behaviors and real-life alcohol consumption levels [26]. One test day was scheduled after an evening of drinking alcohol (the post-alcohol day), the other test day after an alcohol-free day (the control day). The study was conducted at Utrecht University. The University of Groningen Psychology Ethics Committee approved the study, and written informed consent was obtained from all participants.

The aim was to include two groups of social drinkers: (1) N = 18 hangover-sensitive drinkers and (2) N= 18 hangover-resistant drinkers. Allocation was based on a combination of whether or not the participant reported having a hangover after a night of heavy drinking and their score on the one-item hangover severity scale [27]. It was essential that both groups consumed sufficient amounts of alcohol to produce a hangover per se. Therefore, their peak estimated BAC for their usual drinking occasions had to be higher than 0.08%. This was determined by asking participants how many alcoholic units they usually consumed within a certain time frame. By using a modified Widmark formula [28], which takes drinking time and the amount of alcohol consumed into account and controls for sex and body weight, peak estimated BAC was calculated. Notably, in the Netherlands, standard units of alcoholic drinks each contain 10 g alcohol, independent of the type of alcoholic beverage.

To be included, participants had to be healthy (i.e., no physical or mental disease), 18 to 30 years old, non-smoking, not using illicit or medicinal drugs (except contraception), and have received no recent vaccinations. Participants were considered healthy if they reported the absence of physical and mental health conditions and did not receive any pharmacological or psychological treatment. During test days, participants were not allowed to take any treatments to prevent or relieve hangover symptoms, medication that may have an impact on immune functioning, such as acetaminophen, aspirin, and non-steroidal anti-inflammatory drugs (NSAIDs), or to consume caffeinated beverages. Participants were excluded if they reported acute inflammation (infections, common cold, severe acne, flu), allergic reactions (asthma and food allergy), autoimmune diseases (rheumatoid arthritis, multiple sclerosis, diabetes type II), inflammatory bowel disease (Crohn’s disease, ulcerative colitis, irritable bowel syndrome), or other conditions that may have an impact on cytokine concentrations (e.g., chronic fatigue syndrome and fibromyalgia). 

During the training day, demographic data (sex, age, weight, and height) were collected. One of the test days was scheduled after an evening of drinking alcohol (the post-alcohol day), the other after an alcohol-free day (the control day). There were no instructions given with regard to when or how much alcohol to consume. Although a test day was scheduled, subjects could voluntarily cancel the test day if they (also last-minute) did not want to consume alcohol on that day. Then, an alternative test day was scheduled. At screening, and on each test day, a urine drug screen (AlfaScientic Designs, Inc, Poway, CA, USA) was conducted to verify the absence of illicit drug use (including amphetamines (including 3,4-Methylenedioxymethamphetamine, MDMA), barbiturates, cannabinoids, benzodiazepines, cocaine, and opiates). Females were tested for pregnancy. In addition, a breath alcohol test was performed on each test day, using an Alcotest 7410 Breath Alcoholmeter (Dräger, Hoogvliet, The Netherlands). 

At the start of both test days, a saliva sample was collected by the passive drool method in 2-mL polypropylene cryovials, using SalivaBio’s Saliva Collection Aid (Salimetrics, State College, PA, USA). For each subject, on the post-alcohol day and the control day, it was aimed to collect the saliva samples at the same time of day (between 9 and 12 a.m.), in order to prevent circadian influences on cytokine concentrations. Participants were not allowed to eat or drink for at least 30 min before donating the saliva. The cryovials were stored at a temperature of −80 °C.

Saliva cytokine concentrations were determined by multiplex immunoassays (customized Bio-Plex^®^ Multiplex Immunoassay System, BioRad Laboratories, Veenendaal, The Netherlands). All incubations were conducted at room temperature. First, 25 µL of each saliva sample was pipetted into a dilution plate; 25 µL of assay buffer was added to dilute the samples (×2). The magnetic beads were prepared by dilution with 4× buffer and pipetted into a 96-well plate. After washing the beads twice, the standards and the samples were added to the plate and incubated for 30 min. The plate was washed twice and diluted biotinylated detection antibody was added, which was then incubated for 30 min. The plate was washed twice and diluted streptavidin– phycoerythrin (used as detection substrate) was added and incubated for 10 min. The plates were washed twice and assay buffer was added to each well. Fluorescence was read within 30 min.

Single assessments of the following cytokines were made: IL-1β, IL-2, IL-4, IL-5, IL-6, IL-8, IL-10, granulocyte–macrophage colony-stimulating factor (GM-CSF), interferon-gamma (IFN-γ) and tumor necrosis factor-alpha (TNF-α). Cytokine concentrations were expressed in pg/mL saliva. For each cytokine determination, each multiplex plate had a unique lower limit of detection (LOD). For those cytokine concentrations below the LOD of the assay, the LOD value was divided by 2 to enable inclusion of these assessments in the statistical analyses. If more than 25% of the cytokine assessments were below the LOD value, the results for that cytokine were considered to be unreliable and not used for final data analysis.

Overall hangover severity (a one-item hangover score), as well as the severity of 23 individual symptoms, such as nausea, headache, tiredness, and apathy, were assessed on an 11-point scale, ranging from 0 (absent) to 10 (extreme) [25,27].

Statistical analyses were performed using IBM Statistical Package for the Social Sciences (SPSS), version 27. Mean and standard deviation (SD) were computed for all parameters. Cytokines for which more than 25% of determinations were below the LOD were not considered in the statistical analysis. Cytokine data were not normally distributed, and nonparametric tests were used for statistical analyses. The cytokine concentrations and the overall hangover severity on the post-alcohol day and control day were compared for all the participants, as well as for the hangover-sensitive and hangover-resistant groups separately, using the nonparametric related-samples Wilcoxon signed rank test. Applying the nonparametric independent-samples Mann–Whitney U test, data were compared between the hangover-sensitive group and the hangover-resistant group. Difference scores (Δ, post-alcohol day—control day) in cytokine concentrations were correlated with difference scores of severity of both overall hangover and the 23 individual hangover symptoms, using nonparametric Spearman’s rho correlations. *p*-values were adjusted for multiple comparisons (dividing 0.05 by the number of comparisons made, to be used as significance cut-off).

## 3. Results

N = 36 participants completed the study. One hangover-sensitive subject was excluded based on having a much greater alcohol intake when compared to the group average (32 versus 11.1 alcoholic drinks). The final dataset for statistical analysis included N = 17 hangover-sensitive drinkers and *N* = 18 hangover-resistant drinkers. Their demographic data are summarized in Table 1. 

The hangover-sensitive group (*N* = 17, 70.6% female) and the hangover-resistant group (*N* = 18, 55.6% female) did not significantly differ in any demographics, including the number of alcoholic units consumed on the alcohol day and corresponding estimated BAC on the evening before the test day. In the morning of the test days, breath alcohol content was determined. On the alcohol-free test day, breathalyzer assessments were zero in all subjects; on the post-alcohol test day, *N* = 3 subjects tested positive when entering the institute, and their assessments were postponed until BAC reached zero. 

The mean (SD) post-alcohol day saliva collection time was 10.56 a.m. (1.1), which was an average of 9.01 (1.4) hours after cessation of drinking. The control day collection time of 10.37 a.m. (1.1) did not significantly differ (*p* = 0.21) from the post-alcohol day. Table 2 summarizes the mean (SD) saliva cytokine concentrations as assessed on the post-alcohol day and the control day for all participants.

For a number of participants, it was not possible to reliably determine saliva cytokine concentrations. If, for more than 25% of participants, an assessment was below the LOD, either on the post-alcohol day or the control day, these cytokines were omitted from the analyses. This was the case for IL-2, IL-4, IL-5, GM-CSF, and IFN-γ. For several cytokines, an increase in concentration was seen on the post-alcohol day (IL-6, IL-10, and TNF-α). After adjusting for multiple comparisons, significant differences (*p* < 0.01) between the post-alcohol day and the control day were found for IL-6 and Il-10, whereas the difference for TNF-α approached significance. The differences for IL-1β and IL-8 were not significant.

Table 3 summarizes the mean (SD) saliva cytokine concentrations as assessed on the post-alcohol day and the control day for both groups separately. For most cytokines, an increase in concentration was seen on the post-alcohol day. In the hangover-resistant group, the increase reached significance for IL-6 (*p* = 0.005), and the difference in IL-10 approached significance (*p* = 0.011). No other comparisons revealed significant differences after correcting for multiple comparisons.

**Figure 1:**
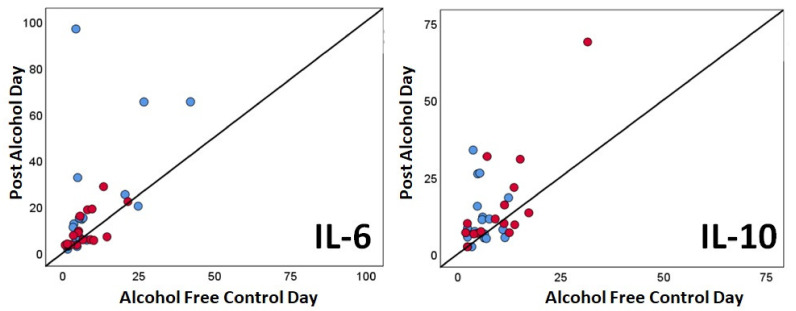
Mean (SD) saliva cytokine concentrations. Individual subject data from the hangover-sensitive group (red markers) and hangover-resistant group (blue markers) are shown for interleukin (IL)-6 and IL-10, which were both significantly increased on the post-alcohol day.

Individual subject data for IL-6 and IL-10 are also summarized in Figure 1. From Figure 1, it is evident that most individuals had higher cytokine concentrations on the post-alcohol day (i.e., above the black diagonal line representing equal concentrations).

To further investigate the magnitude of cytokine changes after alcohol consumption, delta saliva concentrations (Δ, post-alcohol day—control day) for cytokine concentrations were calculated and compared between the hangover-sensitive group and the hangover-resistant group. No significant differences were found between the two groups for any of the cytokines. Furthermore, when comparing the raw data from the control day and post-alcohol day separately, no significant differences were found between the groups.

Hangover-resistant drinkers reported an overall hangover severity score of zero (N = 12) or one (N = 6), resulting in a mean (SD) severity score of 0.3 (0.5). The mean (SD) overall hangover severity score in hangover-sensitive drinkers was 5.9 (2.0). On the control day, the overall hangover severity score was zero (0.0) for both groups. There were no significant correlations between Δoverall hangover severity and changes in cytokine concentrations. Regarding the Δseverity scores of 23 individual hangover symptoms, after correcting for multiplicity (*p* < 0.002), no significant associations were found between Δsymptom severity scores and Δcytokine concentrations. Correlations between ΔIL-6 and Δheadache (r = 0.572, *p* = 0.017) and between ΔIL-6 and Δconcentration problems (r = 0.536, *p* = 0.027) showed a trend towards significance.

## 4. Discussion

This study demonstrated that the immune system is activated after alcohol consumption at levels which produce hangover, as significant increases in salivary cytokine concentrations IL-6 and IL-10 were found during the post-alcohol day. 

Surprisingly, the changes in cytokine concentrations did not significantly differ between hangover-sensitive and hangover-resistant drinkers. In contrast, a clear difference was seen in the presence and severity of hangover symptoms in the hangover-sensitive group and their absence in the hangover-resistant group. This suggests that the latter experienced less intense or no hangover symptoms despite activation of immune responses. Of course, there are various other physiological factors which are involved in hangover [29,30,31,32], but the relative contribution of these to hangover and hangover resistance remains to be determined. Another possibility is that that hangover resistance is actually a manifestation of these drinkers being less aware of their hangover symptoms. Future research might therefore investigate the extent of alexithymia and poor meta-cognition in this population.

Kim et al. [18] reported significant correlations between blood ΔIL-12 and ΔIFN-γ concentrations and changes in total hangover scale scores. Significant correlations with subjective hangover scale scores were found for ΔIFN-γ, and significant correlations with somatic hangover scale scores were found for ΔTNF-α and ΔIL-12. Kim et al. [19] reported significant correlations between blood IL-6 and IL-10 concentrations and hangover severity. In the current study, we did find significant correlations between changes in saliva cytokine concentrations and overall hangover severity. Moreover, for the 23 individual hangover symptoms, no significant correlations with cytokine concentrations were found.

There are several distinctions between the three studies that may account for the observed differences. The most important one is the fact that, in the current study, assessments were performed using saliva, whereas the studies by Kim et al. [18] used stimulated blood cells, and Kim et al. [19] performed assessments on normal blood samples. Further, in the current study and in Kim et al. [18], change scores (hangover–placebo) were used for the correlations, whereas in Kim et al. [19], absolute blood cytokine concentrations were correlated with hangover severity. Various methodological differences between the three studies may also account for the inconsistent findings. First of all, cytokines were assessed at different time points (approximately 9 h after drinking cessation in the current study, versus 12–14 h in the studies by Kim et al. [18], Kim et al. [19], and Mammen et al. [20]). Secondly, these studies included only male participants, whereas the current study included both sexes. In addition, the method of assessing overall hangover severity differed between the studies. Finally, in the studies by Kim et al. [18], Kim et al. [19], and Mammen et al. [20], participants were given a pre-calculated mixture of alcohol in the lab, which was consumed within a set time. In the current naturalistic study, participants were allowed to drink any type of alcohol and non-alcoholic drink at a location and pace of their own choice. This resulted in a mean (SD) drinking time of 5.4 (1.9) hours, which was longer than in the controlled studies, and also resulted in significantly higher estimated BAC levels.

Our findings are in line with other observations which have been published elsewhere [25]. These showed no significant differences between the hangover-sensitive and -resistant drinkers in demographics, alcohol consumption patterns, and mood [25]. Moreover, the groups did not differ significantly in scores on the Alcohol Use Disorders Identification Test (AUDIT) and Self-Rating of the Effects of alcohol (SRE) scale, suggesting no difference in harmful alcohol consumption patterns [25]. Further, there were no differences in reported behaviors during the alcohol consumption sessions (e.g., dancing, sitting in a bar). Morning assessments of urine methanol [33] and urine ethyl glucuronide (EtG) and ethyl sulfate (EtS) [34] did not reveal any significant differences between the hangover-sensitive and -resistant groups. However, urine ethanol concentration was significantly lower in the hangover-resistant group when compared to the hangover-sensitive group [35], suggesting accelerated alcohol metabolism among hangover-resistant drinkers. Although a study that further explored potential differences in alcohol metabolism between hangover-sensitive and -resistant drinkers found no significant differences in breath alcohol concentration, subjective sleepiness, and subjective intoxication after an acute alcohol challenge (BAC 0.05%) [36], another study confirmed that drinkers with a faster ethanol elimination rate experienced less severe hangovers [37]. Future research into alcohol metabolism is warranted to further investigate potential differences between hangover-sensitive and -resistant drinkers. Exploring possible genetic differences related to immune functioning and alcohol metabolism may help to elucidate why some drinkers claim to be hangover-resistant while others are hangover-sensitive. 

The use of a naturalistic study design has many strengths [26]. As opposed to controlled trials, this design closely mimics a real-life drinking session. Type and quantity of beverage consumption are under control of the subjects only, as well as the chosen venues and their behaviors (e.g., talking, dancing). Alcohol consumption levels are usually higher than allowed in controlled studies [25], better reflecting their normal drinking behavior that provokes hangovers. Characteristic of the naturalistic study design is the fact that researchers are not present during the drinking session and thus cannot interfere with the subjects’ natural behaviors. Important information, such as the amount of alcohol consumed, is retrospectively reported to the investigators. From these data, important study variables are estimated and calculated, such as the peak BAC. Assessments during the hangover state are made in the presence of the investigators. 

The study also has some limitations that should be addressed. First of all, a disadvantage of the naturalistic approach is that it has to rely in part on retrospective reporting, while it may be preferable to monitor some of the parameters very carefully [26]. The timing of the saliva specimen would then have been exact. Moreover, some other variables which showed large interindividual differences, such as the start and stop time of alcohol consumption or time to bed, could be kept stable across subjects in controlled studies. Another example of an interindividual difference is food intake during the drinking session. In the current study, this was assessed in an unsystematic way, not allowing analysis. However, from the collected data, it can be concluded that food intake differed between the participants. Previous research showed that food intake and specific nutrients may have an impact on reported hangover severity [38,39]. Therefore, it is advised that future naturalistic studies should collect data on food intake using systematic assessment tools such as food frequency questionnaires. The amount of water and nonalcoholic beverages consumed was not assessed in the current study. Although previous research did not reveal any relationship between the consumption of water and nonalcoholic beverages and hangover severity [40,41], this may have had an effect on cytokine concentrations. Therefore, it is advised to collect data on water and nonalcoholic beverage consumption in future studies. Taken together, it would be useful to replicate the current findings in a controlled trial with preset drinking times and sleeping times, including the same type and amount of alcohol consumption for all subjects. 

Saliva samples were taken in the current study, as this is a noninvasive method which is more friendly for participants when compared to blood drawing. Saliva sample collection has many advantages over blood drawing, including being more cost-effective and not painful for participants. Compared to collecting urine or drawing blood, patients strongly prefer the collection of saliva samples [42,43]. In this study, no blood samples were taken, so a direct comparison to determine whether these matched was not possible. However, the literature shows that saliva assessments closely match results from blood drawings and that both assays are a valid method to gather information on immune functioning and its relationship with emotional states [44,45,46]. Nevertheless, it should be kept in mind that cytokine concentrations are influenced by many external factors. Several of these factors were under control in this study (e.g., the absence of immune-related disease was used as an inclusion criterion), while others were systematic interpersonal differences which were stable within subjects (e.g., oral health, and time of day that the saliva samples were taken on the post-alcohol day and control day), while other factors that may influence cytokine concentrations were the variables under investigation that were deliberately uncontrolled in this naturalistic study (e.g., time after drinking, peak estimated BAC).

In line with previous research, we chose to assess cytokines as biomarkers of the innate immune response. The purpose of the innate immune response is to immediately protect the body against foreign pathogens and toxics such as alcohol. In contrast, adaptive immunity is acquired over a lifetime after previous exposure to a toxic, to prepare the body’s immune system for future challenges. Repeated alcohol abuse has been associated with a reduction in the number of B and T cells [47], which are involved in adaptive immunity. Therefore, in future research, it may be of interest to assess biomarkers of the adaptive immune response, including B cells and T cells. 

Finally, future research should include measurements at multiple time points during the day. It has been previously reported that measuring hangover severity at a single time point on both test days is a limitation as temporal fluctuation of presence and severity during the day are not taken into account [48]. Temporal changes in hangover severity have also recently been investigated, showing that different types of severity patterns occur across drinkers [49]. These different patterns might explain the absence of significant associations with changes in cytokine concentrations, as, in the current study, participants were not selected based on their temporal hangover severity profile. Concentrations of inflammatory markers may also fluctuate throughout the day [50,51], which makes it important to at least standardize the time of sample collection across test days. To better capture the circadian effects of both cytokine concentrations and hangover severity, in future research, having multiple assessment points during the day is warranted. 

## 5. Conclusions

The current findings confirm that the immune system responds to alcohol consumption, which is measurable on the post-alcohol day. As the observed cytokine changes do not differ significantly between hangover-sensitive and -resistant drinkers, and they do not significantly correlate with overall hangover severity or individual symptom severity, the exact nature of the association between immune functioning and the hangover state warrants further investigation.

## Figures and Tables

**Table 1:** Demographics.

Demographics	All Participants	Hangover-Resistant	Hangover-Sensitive	*p*-Value
N	35	18	17	
Male/female	13/22	8/10	6/11	
Age (year)	21.1 (1.8)	20.8 (2.0)	21.4 (1.6)	0.356
Height (m)	1.77 (0.1)	1.78 (0.1)	1.76 (0.1)	0.397
Weight (kg)	68.5 (10.3)	71.1(10.2)	65.8 (1.0)	0.126
BMI (kg/m^2^)	21.9 (2.2)	21.4 (2.3)	22.3 (2.0)	0.186
Alcoholic units consumed	11.0 (5.1)	10.7 (4.7)	11.3 (5.6)	0.715
Estimated BAC (%)	0.17 (0.7)	0.17 (0.07)	0.17 (0.07)	0.701

Mean and standard deviation (SD) are shown. Abbreviations: BAC = blood alcohol concentration, BMI = body mass index.

**Table 2:** Mean (SD) saliva cytokine concentrations of all participants.

Test Day	Post-Alcohol Day		Control Day		
Cytokine	Mean (SD)	% Below LOD	Mean (SD)	% Below LOD	*p*-Value
IL-1β	158.7 (205.9)	0.0% (0/35)	198.4 (413.6)	0.0% (0/35)	0.523
IL-6	16.4 (20.5)	2.9% (1/35)	8.6 (8.8)	2.9% (1/35)	0.001 *
IL-8	584.3 (668.8)	0.0% (0/35)	809.3 (1725.0)	0.0% (0/35)	0.342
IL-10	13.7 (12.9)	5.7% (2/35)	7.8 (5.9)	17.1% (5/35)	0.001 *
TNF-α	85.2 (51.1)	0.0% (0/35)	70.6 (46.5)	0.0% (0/35)	0.038

Mean (pg/mL) and standard deviation (SD) are shown for all N = 35 participants. Abbreviations: LOD = lower limit of detection, IL = interleukin, TNF-α = tumor necrosis factor-alpha. Cytokines with more than 25% of participants below LOD are not shown. Significant differences (*p* < 0.01, after adjusting for multiple comparisons) between the post-alcohol and control day are indicated by *.

**Table 3:** Mean (SD) saliva cytokine concentrations.

	Hangover-Resistant Group (N = 18)			Hangover-Sensitive Group (N = 17)		
Cytokine	Post-Alcohol Mean (SD)	Control Day Mean (SD)	*p*-Value	Post-Alcohol Mean (SD)	Control Day Mean (SD)	*p*-Value
IL-1β	179.4 (196.8)	148.1 (237.2)	0.732	136.7 (219.0)	251.6 (545.8)	0.981
IL-6	22.4 (26.6)	9.8 (11.2)	0.005 *	10.1 (7.6)	7.4 (5.4)	0.084
IL-8	582.3 (614.6)	626.7 (1004.7)	0.472	586.4 (741.0)	1002.7 (2274.6)	0.523
IL-10	12.0 (8.8)	6.0 (3.1)	0.011	15.4 (16.2)	9.8 (7.6)	0.041
TNF-α	79.6 (42.2)	62.9 (38.3)	0.048	91.2 (59.8)	78.8 (53.8)	0.332

Mean (pg/mL) and standard deviation (SD) are shown. Significant differences (*p* < 0.01, after adjusting for multiple comparisons) between the post-alcohol day and the control day are indicated by *. No significant differences were found for any of the assessments between hangover-sensitive and -resistant drinkers.

## Data Availability

The data are available upon request from the corresponding author.
